# Phage Display-Derived Compounds Displace hACE2 from Its Complex with SARS-CoV-2 Spike Protein

**DOI:** 10.3390/biomedicines10020441

**Published:** 2022-02-14

**Authors:** Marc Sevenich, Elena Thul, Nils-Alexander Lakomek, Thomas Klünemann, Maren Schubert, Federico Bertoglio, Joop van den Heuvel, Patrick Petzsch, Jeannine Mohrlüder, Dieter Willbold

**Affiliations:** 1 Institute of Biological Information Processing (IBI-7), Forschungszentrum Jülich, 52425 Jülich, Germany; m.sevenich@fz-juelich.de (M.S.); n.lakomek@fz-juelich.de (N.-A.L.); 2 Priavoid GmbH, 40225 Düsseldorf, Germany; 3 Medizinische Fakultät, Westfälische Wilhelms Universität Münster, 48149 Münster, Germany; e_thul01@uni-muenster.de; 4 Institut für Physikalische Biologie, Heinrich-Heine-Universität Düsseldorf, 40225 Düsseldorf, Germany; 5 Helmholtz Centre for Infection Research, 38124 Braunschweig, Germany; thomas.kluenemann@helmholtz-hzi.de (T.K.); joop.vandenheuvel@helmholtz-hzi.de (J.v.d.H.); 6 Institut für Biochemie, Biotechnologie und Bioinformatik—Abteilung Biotechnologie, Technische Universität Braunschweig, 38106 Braunschweig, Germany; m.bleckmann@tu-bs.de (M.S.); f.bertoglio@tu-bs.de (F.B.); 7 Biologisch-Medizinisches Forschungszentrum (BMFZ), Heinrich-Heine-Universität Düsseldorf, 40225 Düsseldorf, Germany; patrick.petzsch@uni-duesseldorf.de; 8 JuStruct, Forschungszentrum Jülich, 52425 Jülich, Germany

**Keywords:** SARS-CoV-2, coronavirus, spike protein, phage display, acute COVID-19 treatment, spike-hACE2 inhibitor

## Abstract

Severe respiratory syndrome coronavirus-2 (SARS-CoV-2) is a highly contagious beta-class coronavirus. Although vaccinations have shown high efficacy, the emergence of novel variants of concern (VOCs) has already exhibited traits of immune evasion. Thus, the development of tailored antiviral medications for patients with incomplete, inefficient, or non-existent immunization, is essential. The attachment of viral surface proteins to the cell surface is the first crucial step in the viral replication cycle, which for SARS-CoV-2 is mediated by the high affinity interaction of the viral trimeric spike with the host cell surface-located human angiotensin converting enzyme-2 (hACE2). Here, we used a novel and efficient next generation sequencing (NGS) supported phage display strategy for the selection of a set of SARS-CoV-2 receptor binding domain (RBD)-targeting peptide ligands that bind to the target protein with low µM to nM dissociation constants. Compound CVRBDL-3 inhibits the SARS-CoV-2 spike protein association to hACE2 in a concentration-dependent manner for pre- as well as post-complex formation conditions. Further rational optimization yielded a CVRBDL-3 based divalent compound, which demonstrated inhibitory efficacy with an IC_50_ value of 47 nM. The obtained compounds were not only efficient for the different spike constructs from the originally isolated “wt” SARS-CoV-2, but also for B.1.1.7 mutant trimeric spike protein. Our work demonstrates that phage display-derived peptide ligands are potential fusion inhibitors of viral cell entry. Moreover, we show that rational optimization of a combination of peptide sequences is a potential strategy in the further development of therapeutics for the treatment of acute COVID-19.

## 1. Introduction

SARS-CoV-2 is a beta class coronavirus with the ability for human-to-human transmission that was initially discovered and characterized in Wuhan, China, at the end of 2019 [1,2]. Since then, it has challenged health care systems due to its rapid spread and the transmission of COVID-19 throughout the world. Far more than the past coronavirus pandemics, caused by SARS-CoV in 2002 and MERS-CoV in 2012, the ongoing pandemic has claimed over 5.7 million lives with over 386 million total cases across the world [3,4,5,6]. 

The coronavirus replication depends on a multi-step process starting with the interaction of the viral trimeric spike protein (S) and the human angiotensin converting enzyme (hACE2) receptor that mediates uptake of the viral RNA into the host-cell cytoplasm. Each monomer of the spike protein consists of the two functional subunits S1 and S2. The S1 subunit comprises the receptor binding domain (RBD) that interacts via a defined motif sequence (RBM) with an N-terminally located helical structure of the hACE2. In accordance with previous observations from SARS-CoV, the SARS-CoV-2 RBD has been found to exist in two conformational states, from which the ‘up’ state initiates interaction with hACE2 and the ‘down’ state’s main function is the shielding of receptor binding relevant proportions from immune reactions [7]. The S2 subunit plays a crucial role in the membrane fusion process. While the S1-hACE2 interaction allows viral attachment to the host cell surface, the S2 site is cleaved by the human endoprotease TMPRSS2. Together with cooperative actions of other tissue specific endogenous proteases, proteolytic cleavage results in irreversible conformational changes of the S protein that eventually results in cell membrane fusion and endocytic host-cell uptake [8].

Although the viral S proteins of SARS-CoV and SARS-CoV-2 share ~73% amino acid sequence identity, SARS-CoV-2 shows significantly enhanced transmission efficiency when compared to SARS-CoV [9,10]. Structural studies suggest that alterations at the furin cleavage site, located at the boundary between S1 and S2 subunits, may result in reduced stability of the S protein, which facilitates the conformational adaptation that is required for receptor binding [11]. In addition, SARS-CoV-2 specific amino acid residues identified within the RBM were shown to enhance the binding affinity of the SARS CoV-2 spike protein and hACE2 as compared to SARS-CoV [12,13,14]. 

Since its first appearance in 2019, the virus has undergone numerous mutational events, resulting in variants of concern (VOCs) with enhanced fitness regarding their transmissibility and disease severity [15,16]. A large proportion of these mutations cluster in the spike protein, influencing its binding affinity towards hACE2 [13,17,18,19,20]. To date, the most widespread mutants such as B.1.617.2 (δ-variant), P.1 (γ-variant) and B.1.1.529 (ο-variant) have widely replaced the originally identified SARS-CoV-2 virus due to their enhanced fitness [21,22,23,24,25]. Interestingly, most of the circulating variants that have enhanced infectivity as compared to the ancestral strain carry a D614G substitution in the S1 domain of the spike protein. Several indications suggest that the mutation may be directly linked to the enhanced transmissibility [21,24]. 

Although mRNA-based vaccines in particular turned out as a successful strategy for controlling the pandemic spread of SARS-CoV-2, vaccinations have already been shown to be partially less effective for certain VOCs [19,25]. The selection pressure created by different immunization strategies in combination with naturally occurring spontaneous mutations consequently leads to the enforcement of variants with increased immune evasive traits, making adaptation of vaccines as well as repetitive renewal of immunization mandatory. This situation, which resembles the status quo of the influenza virus [26], requires the development of antiviral medication, especially for cases where either no sufficient immunity is present or already existing immunity is circumvented by novel viral strains. 

Hence, various research efforts have either focused on the development of novel drug candidates for a causative treatment such as Paxlovid (Pfizer), repurposing approved compounds such as Molnupiravir (Merck), or even screening components from natural sources [27,28,29,30,31,32,33]. However, there are no generally proven effective antiviral treatment options for SARS-CoV-2 infections. In this study, we present a novel phage display based strategy for the development of SARS-CoV-2 RBD-targeting peptide ligands. The resulting lead compounds show high binding affinity for the RBD in the nanomolar scale and binding selectivity for the SARS-CoV-2 RBD, as well as for the B.1.1.7 mutant (α-variant). Moreover, when analyzed in binding experiments with the isolated proteins, one of the selected peptide sequences reduced the SARS-CoV-2 spike protein binding towards the cellular receptor hACE2 when added before complex formation on one side, but was also able to displace the spike protein from the already formed complex. In addition, a first optimization of the most promising lead compound resulted in an even more efficient compound.

## 2. Materials and Methods

### 2.1. Phage Display Selection

In phage display, exogenous peptides are presented on phage particles as fusion constructs with coat proteins. Consecutive rounds of biopanning and amplification increase the fraction of phages presenting strong target binders, which can be identified by sequencing of the variable portion of the genome [34]. 

For biopanning, the commercially available M13-bacteriophage library TriCo-16 (Creative Biolabs, Shirley, USA) was used. The library had a capacity of 2.6 × 10^10^ pIII fused 16-mer peptide variants. The biotinylated SARS-CoV-2 RBD target protein (positions 319 to 541) was purchased as lyophilized powder from Acrobiosystems (# QHD43416, USA) with a purity of >95%. The protein carries a C-terminal His-Tag for Ni-NTA purification followed by an Avitag sequence (SPD-C82E9, [35]) for BirA catalyzed transfer of biotin. The protein was expressed in HEK293 cells and had a Mw of 28.2 kDa with additional 5 to 8 kDa due to glycosylation. For surface immobilization, the target protein was incubated on a streptavidin-functionalized polystyrene 96-well plate (High capacity, Thermo Fisher Scientific, Waltham, MA, USA) for 1 h at RT using 70 pmol/well of target protein in 25 mM HEPES, pH 7.4, 150 mM NaCl, 100 µM EDTA (selection buffer). Non-coupled streptavidin was quenched by 10 min incubation with 100 µL of 10 µM biotin. Subsequent surface blocking was performed for 1 h by incubation with either 1% (*w*/*v*) BSA or nonfat milk powder in selection buffer. For the first selection round, 10 µL of the phage library (6 × 10^11^ phages/well) was diluted in 90 µL selection buffer with 0.2% (*w*/*v*) BSA or milk powder and incubated for 20 min. The subsequent washing steps with 200 µL washing buffer (0.05% (*v*/*v*) Tween-20 and either 0.2% (*w*/*v*) bovine serum albumin (BSA) or milk powder in selection buffer) were gradually increased from 5 to 15 repetitions with the number of selection rounds. Phage elution was performed using 10 min incubation with 100 µL of 0.2 M glycine-HCl (pH 2.2), followed by neutralization in 25 µL of 1 M Tris-HCl (pH 9.1). Eluted phages were amplified for 4.5 h in 20 mL of LB-cultured *E. coli* K12 ER2783 with a starting OD_600_ of 0.1. The pelleted cells were discarded after 20 min centrifugation at 5000× *g* and the phages were precipitated by addition of 7 mL 20% (*w*/*v*) PEG-8000, 2.5 M NaCl followed by overnight (ON) incubation at 4 °C. Phages were pelleted by centrifugation at 5000× *g* for 1 h at 4 °C and resuspended in 1 mL PBS (pH 7.4). After additional centrifugation at 10,000 rpm for 10 min, the supernatant was mixed with 200 µL of 20% (*w*/*v*) PEG-8000, 2.5 M NaCl and incubated for 1 h at 4 °C. The phages were pelleted in a last purification step for 1 h at 5000 rpm and the pellet was suspended in 100 µL selection buffer. Phage concentration of the purified phages was determined by serial dilution of the phage suspension and incubation with *E. coli* K12 ER2783 on IPTG/X Gal-LB titer plates ON at 37 °C. The concentration of input phages was calculated based on the serial dilution and a concentration of 6 × 10^12^ phage forming units ml^−1^ (PFU) was used as the input for all selection rounds and controls. The selection was performed for three consecutive rounds on the target (target selection = TS). Additionally, input phages resulting from selection rounds on the target were applied to a parallel selection (direct control = DC) on a surface without target, which was otherwise treated identically to the TS surface. As a second control, a consecutive selection was performed exclusively without target on otherwise identically treated surfaces (empty selection = ES). 

### 2.2. Enrichment ELISA

Enrichment-ELISA is a method to identify enrichment of target binding phages during phage display selection. All steps were carried out at RT. A total of 20 pmol/well of the biotinylated SARS-CoV-2 RBD target protein was immobilized on a streptavidin- functionalized polystyrene 96-well plate (High capacity, Thermo Fisher, USA). Both the target-immobilized and target-free surfaces were quenched with biotin as described previously. The surfaces were blocked with 1% (*w*/*v*) BSA in selection buffer for 1 h and washed with washing buffer (0.2% (*w*/*v*) BSA, 0.05% Tween-20 in selection buffer). A total of 2.5 × 10^11^ phages from the TS input samples or library were diluted in 100 µL washing buffer and incubated on target and control surfaces for 1 h. After washing with 200 µL washing buffer, the anti-M13 gpVIII monoclonal HRP-antibody (Sino Biologicals, Beijing, CHN) was diluted to a final concentration of 0.4 mg mL^−1^ in washing buffer and 100 µL per well was incubated for 1 h. After six-fold washing with washing buffer, 100 µL of 3,3′,5,5′-3,3,5,5-Tetramethylbenzidin (Sigma-Aldrich, St. Louis, MO, USA) substrate solution was incubated for 20 min and stopped by addition of an equal volume of 2 M H_2_SO_4_. The signal intensity was quantified by absorption measurement in a Fluorostar optima platereader at 450 nm (BMG Labtech, Ortenberg, GE; *n* = 3). 

### 2.3. ssDNA Purification and Next Generation Sequencing (NGS) of Phage Samples 

The ssDNA of the phage suspensions resulting from each phage display selection round was purified by phage precipitation, as described before, followed by resuspending in 200 µL of a 10:1 mixture of 3 M sodium acetate pH 5.2:1 × TE-buffer and addition of 500 µL of 99% ethanol. ssDNA was precipitated at 14,000 rpm for 10 min at 4 °C and the supernatant was discarded. After pellet purification by the addition of 250 µL of 70% EtOH and repeated centrifugation at 10,000 rpm, the pellet was dried and the DNA concentration was determined by signal quantification at A_260_. For next generation sequencing, a polymerase chain reaction (PCR) was used to add adapter sequences to both the 3′ and 5′ end of the amplification product. Success of the amplification was determined by agarose gel separation with ethidium bromide staining. Amplicon next generation sequencing was performed with a MiSeq system (Illumina, San Diego, CA, USA).

### 2.4. Analysis of Next Generation Obtained Sequences

Variable DNA sequences resulting from next generation sequencing (NGS) were transcribed into peptide sequences, as described previously [36]. The transcribed sequences were filtered based on their relative frequency increase within the TS (library < TS1 < TS2 < TS3), the relative frequency of a sequence observed in one TS compared to its abundancy within the respective DC (TS2 > DC2; TS3 > DC3) and the relative frequency of one sequence observed in one TS round compared to its abundancy within the respective ES round (TS1 > ES1; TS2 > ES2; TS3 > ES3). Sequences of the last TS round that passed the filter were ranked corresponding to their enrichment from library to TS3 (TS3/library = enrichment factor) and their relative frequencies in TS3 compared to ES3 (TS3/ES3 = empty score). Filtered sequences were used as inputs for the Hammock clustering software [37]. The input FASTA-file included all filtered sequences as ranked by their empty score. The Hammock clustering software was performed in full mode with sequences ranked according to their input position (-R input). Cluster motif logos were generated from initial clusters after greedy clustering using the WebLogo 3.4 application. 

### 2.5. CVRBDL Compounds

CVRBDL peptides were purchased with C-terminal amidation from CASLO (Copenhagen, DK) as lyophilized chloride salt powder with a purity of >95%.

### 2.6. SARS-CoV-2 Spike Protein and hACE2 Sample Preparation

**Figure A1:**
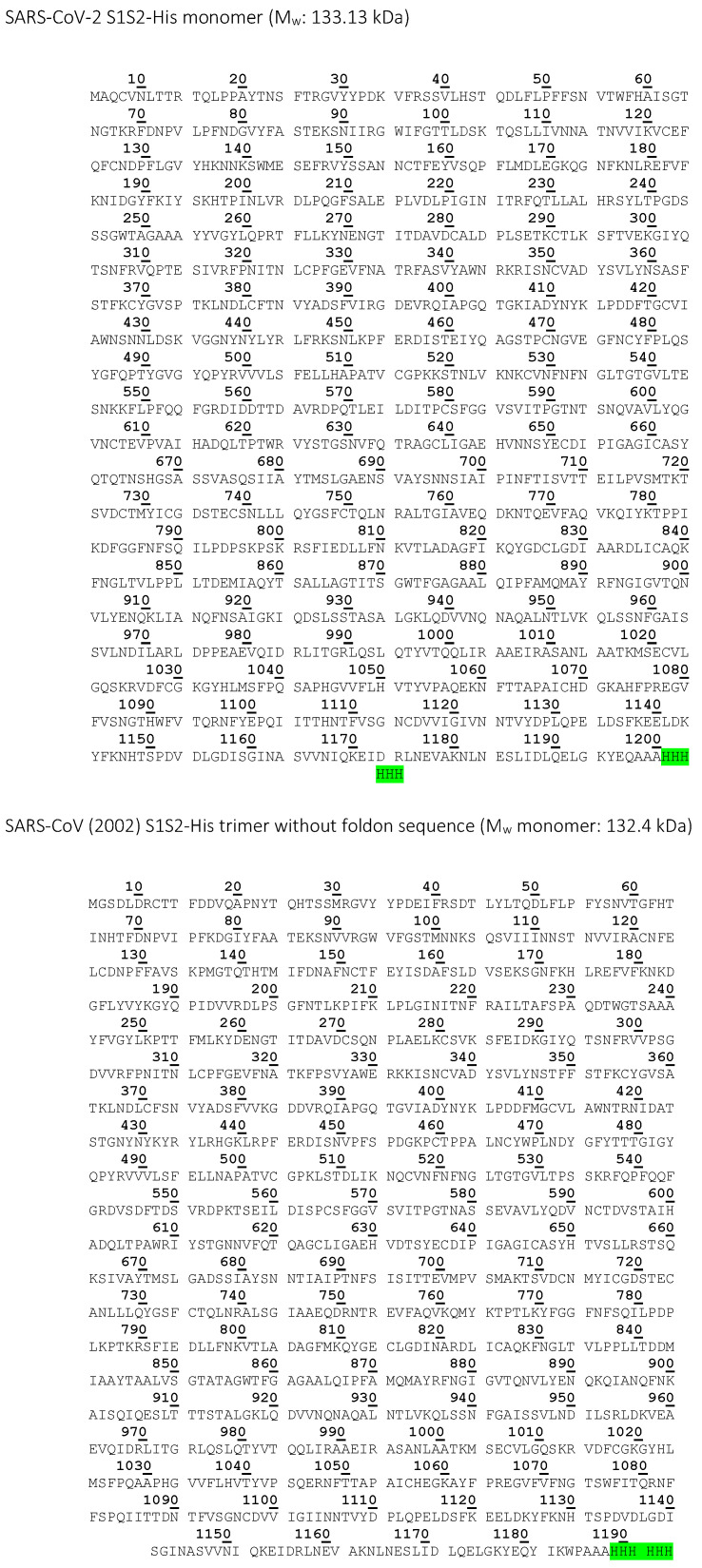

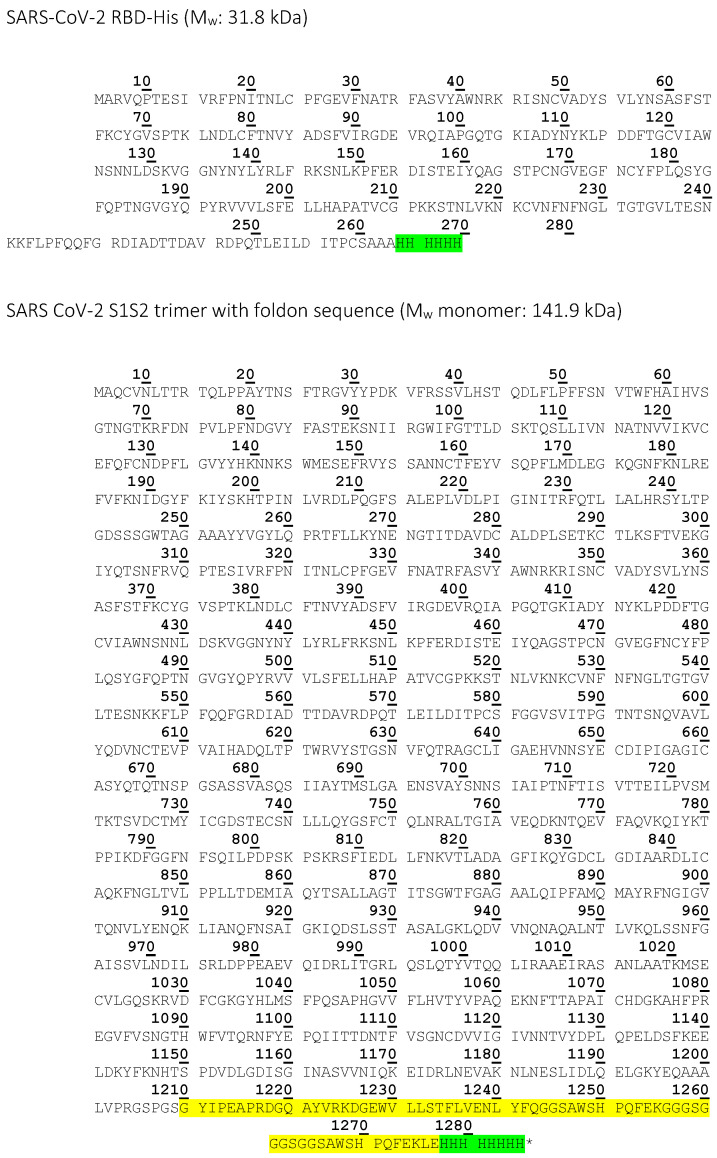

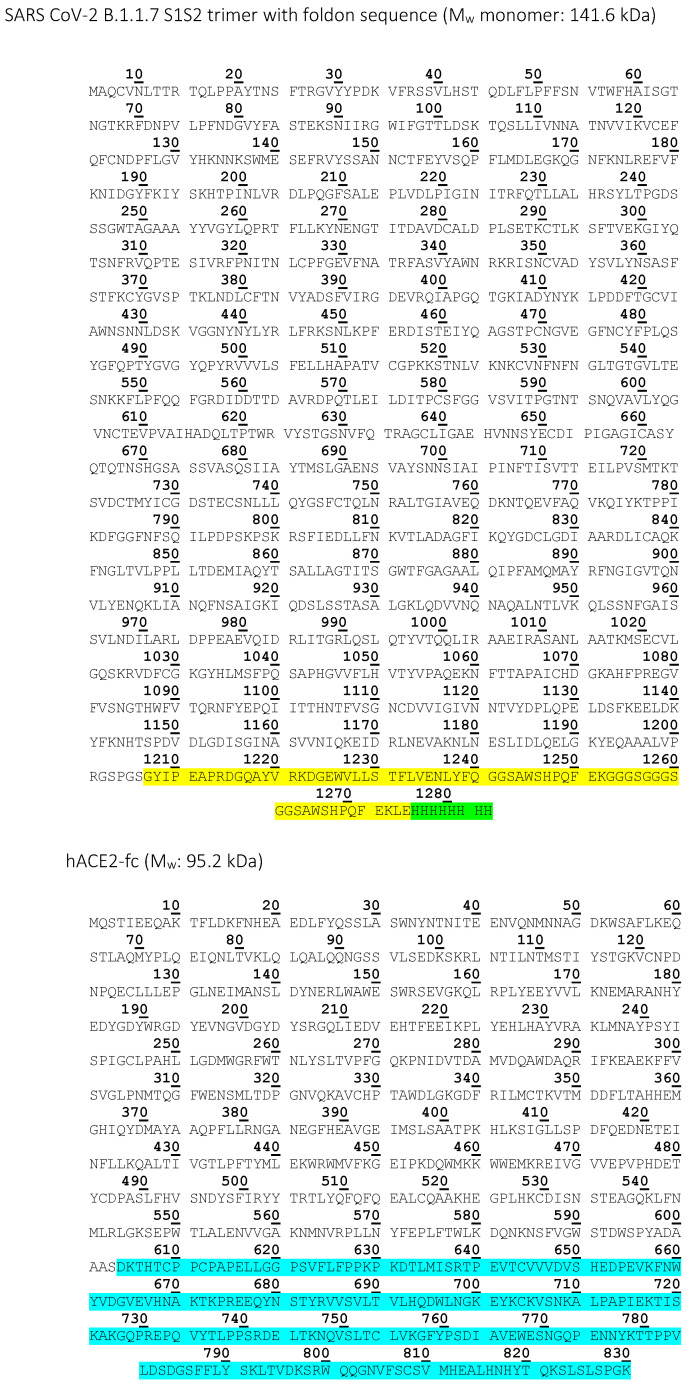
Single letter amino acid sequence of the SARS-CoV spike protein and hACE2 constructs used for SPR experiments. T4 fold-on sequences are marked in yellow, Poly-His sequences are marked in green and fc-tags are marked in turquoise.

Expression of SARS-CoV-2 spike protein constructs was either performed using High Five insect cells (SARS-CoV-2 RBD, SARS-CoV-2 S1S2 monomer and trimer, SARS-CoV-2 B.1.1.7 S1S2 trimer, SARS-CoV (2002) trimer, and SARS-CoV-2 “closed” S1S2 trimer) or HEK293-6E cells (hACE-2) via transient gene expression [38,39]. The High Five (Hi5) insect cell line (officially called BTI-Tn-5B1-4) was isolated by the Boyce Thompson Institute for Plant Research (Ithaca, NY, USA); the cell line can be acquired from Thermo Fisher Scientific (USA). The HEK293-6E cell line was licensed from National Research Council (NRC), Biotechnological Research Institute (BRI, CAN). Recombinant protein genes for the CoV constructs (Figure A1) were synthesized by Genscript (Piscataway Township, NJ, USA) or Thermo Fisher Scientific (Waltham, MA, USA). Protein samples were purified using HisExcel columns (Cytiva, Marlborough, MA, USA) for 6xHis tagged proteins, StrepTrap HP columns (Cytiva, MA, USA) for TwinStrep tagged proteins (all trimeric proteins with fold-on sequences were Strep and His-tagged and purified via StrepTrap HP columns) or protein A columns (Thermo Fisher Scientific, USA) for Fc-tagged proteins (see Table A1 for complete list of all constructs and modifications). A C-terminal introduction of a T4 bacteriophage fold-on sequence (with exception of the SARS-CoV construct) was used for the induction of S1S2 stable trimers [40]. All purifications were performed on Äkta Start or Äkta Pure systems (Cytiva, USA) according to the manufacturer’s protocol. Depending on the protein size, size exclusion chromatography (SEC) was performed as a final polishing step using either a Superdex 200 or Superose 6 column (Cytiva, USA) in 20 mM Tris (pH 8.0), 150 mM NaCl as running buffer. The SARS-CoV-2 nuclear magnetic resonance (NMR) sample was additionally purified via 10/300 Superdex 200 Increase SEC (Cytiva, USA) in 20 mM 2-(*N*-morpholino)ethanesulfonic acid (MES) (pH 6.5), 100 mM NaCl. The oligomerization state and purity of each construct was verified by SEC—multiangle light scattering (SEC-MALS).

### 2.7. NMR Sample Preparation

SARS-CoV-2 RBD protein was expressed in HighFive insect cells as described previously. The NMR sample was prepared using 0.85 mM (25.7 mg/mL) SARS-CoV-2 RBD, dissolved in 20 mM MES (pH 6.5), 100mM NaCl sample buffer. After recording the reference NMR spectrum of the RBD domain alone (see below), CVRBDL-3 was added to a final concentration of 1 mM, resulting in a molar stoichiometry of 1.1:1 for the peptide vs. RBD domain.

### 2.8. NMR Spectroscopy

Two dimensional ^1^H-^15^N HMQC (heteronuclear multiple quantum coherence) spectra of the isolated RBD domain (natural abundance) were recorded on a Bruker Avance Neo 1.2 GHz spectrometer, equipped with a 3 mm HCN cryoprobe. Spectral dimensions were 16.02 ppm (^1^H) × 32.89 ppm (^15^N); 1024 and 27 complex points were recorded in the direct (^1^H) and indirect (^15^N) dimension, resulting in acquisition times of 53.2 ms and 6.8 ms in the direct and indirect dimension, respectively. To compensate for the low sensitivity of the natural abundance sample (^15^N natural abundance of 0.37%), 8192 scans were recorded for each increment in the indirect dimension. The recovery delay between experiments was set to 1 s, resulting in a total experimental time of 5 d 15 h per spectrum. The experimental temperature was 20 °C. Spectra of the RBD domain in the presence of the peptide, as well as for the isolated peptide, were recorded back-to-back, under identical conditions as for the reference spectrum of the RBD domain alone. Spectra were processed using the Bruker Topsin 4 software (Billerica, MA, USA) and analyzed using the CCPN Analysis 2.3 software (Collaborative Computing Project for NMR; [41]).

### 2.9. Surface Plasmon Resonance (SPR) Experiments 

SPR experiments were performed with a Biacore T200 device (Cytiva, USA) using 10 mM HEPES (pH 7.4), 150 mM NaCl, 3 mM EDTA, 0.005% (*v*/*v*) Tween-20 as assay buffer. SPR experiments were performed at a flow rate of 30 µL/min and RT if not otherwise stated.

### 2.10. SPR Affinity Measurements

Affinity screening for CVRBDL peptides −1 to −5 was performed by immobilization of the biotinylated SARS-CoV-2 RBD selection target (Acrobiosystems, Newark, NY, USA) on a streptavidin-functionalized sensor surface (SA sensor, Cytiva, USA). CVRBDL peptides were injected for 300 s in a concentration range of 11.1 to 0.01 µM, followed by 600 s of dissociation phase detection. For affinity measurements on SARS-CoV-2 and COVID (2002) RBD, S1S2 monomer and S1S2 trimer constructs, the proteins were immobilized on a polycarboxylate sensor surface using EDC/sulfo-NHS chemistry at pH 5.5 (HC200M, Xantec, GE). Injections were performed for 60 s association and 100 s dissociation using CVRBDL-3 and -3_3 concentration ranges of 5 to 0.04 µM. For both setups, regeneration was performed using 2 × 30 s injections of 20 mM glycine-HCl (pH 3.5). Data evaluation was performed using Biacore T200 Evaluation Software v3.2 (Cytiva, USA). 

### 2.11. SPR Inhibition Assay 

The SPR inhibition assay was performed by immobilization of hACE-fc on a Protein A functionalized sensor surface by injecting 6 µg/mL protein for 45 s at 10 µL/min (Protein A, Cytiva, USA). Next, 50 nM of the SARS-CoV-2 S1S2 monomer was injected for 300 s with or without compound concentrations in the range of 100 µM to 6.4 nM and 5 µM to 20 nM for CVRBDL-3 and -3_3, respectively. All injections including compound were referenced with the corresponding injections of compound only on the immobilized hACE2, while the S1S2 injection without compound (before and after the concentration row) was referenced with a buffer only injection. For IC_50_ validation, the responses at injection times of t = 750 s were plotted against the log_10_ of the used compound concentration. Data fitting was performed using a Boltzmann fit model (OriginPro 2020, OriginLab, Northampton, MA, USA): y = (A1 – A2)/(1 + e^((x – x_0_)/dx)) + A2 with A1 = initial value, A2 = final value, x_0_ = inflection point = IC_50_, and dx = time constant. 

### 2.12. SPR Displacement Assay 

The displacement assay was performed by hACE2-fc immobilization as described in the previous section. Next, 50 nM SARS-CoV-2 wt S1S2 was injected for 300 s at 30 µL/min, followed by a dissociation recording of 1200 s. During dissociation, different concentrations of CVRBDL-3 and -3_3 were injected in the range of 2 µM to 0.63 µM for 300 s. These injections were referenced with compound-only injections using the same concentration on the immobilized hACE-fc. Data evaluation was performed by plotting the response at t = 1050 s against the corresponding compound concentration. Displacement effects were fitted using a mono exponential decay model (OriginPro 2020, OriginLab, Northampton, MA, USA): y = y_0_ A e^(−x/t) with y_0_ = offset, A = amplitude, t = time constant, and t ½ = DC_50_ = t∙ln(2).

## 3. Results

### 3.1. Phage Display Selection on the SARS-CoV-2 RBD Target Protein

**Figure 1:**
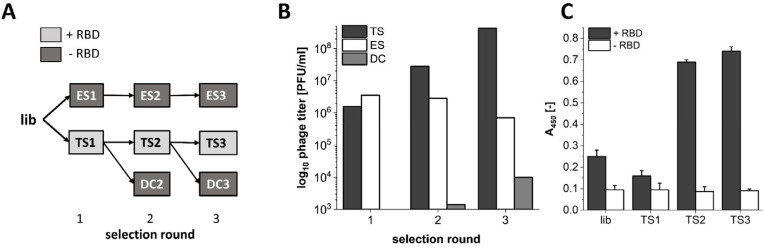
M13 phage display selection on SARS-CoV-2 RBD in three consecutive selection rounds. (**A**) Selection scheme: lib = naïve library; TS = target selection on RBD; DC = direct control; ES = empty selection. Samples are derived from each other in the direction of the connecting arrow starting with the naïve phage display library (lib). For TS, the selection target SARS-CoV-2 RBD was immobilized on a streptavidin-functionalized surface, while no target protein was immobilized for ES and DC. (**B**) Output phage titer in PFU/mL for selection samples of TS, ES and DC as measured past phage elution after biopanning. (**C**) Enrichment ELISA with input titer samples on the immobilized SARS-CoV-2 RBD selection target protein. Phage concentration was adjusted based on A_265_ UV-vis measurements to a total input of 2.5 × 10^10^ phages for all samples.

For identification of RBD binding peptide sequences, M13 phage display selection was performed on the biotinylated SARS-CoV-2 RBD target protein in three consecutive selection rounds (TS = target selection). The control selections on “empty” surfaces (same surfaces but without RBD) were either derived from selection rounds on the target (DC = direct control) or from a selection starting from the original library that was exclusively performed without target protein (ES = empty selection; Figure 1A). 

During the selection process, the success of target-related sequence enrichment was verified by determination of the PFU concentration in phage elution suspensions after biopanning (Figure 1B). Here, a 270-fold increase of the output titer was observed for the TS3 as compared to TS1, while an output titer reduction by a factor of ~5 was observed when no target was present during biopanning (ES3 compared to ES1). Likewise, the titer of TS derived samples decreased when a surface without target was used for the subsequent selection round (DC). In addition, the enrichment ELISA with the input titer phage samples showed a ~5-fold increase in end-point signal on the selection target, while a low signal was identified when no selection target was present (Figure 1C). 

### 3.2. Analysis of Sequences Derived from NGS Sequencing 

**Figure 2:**
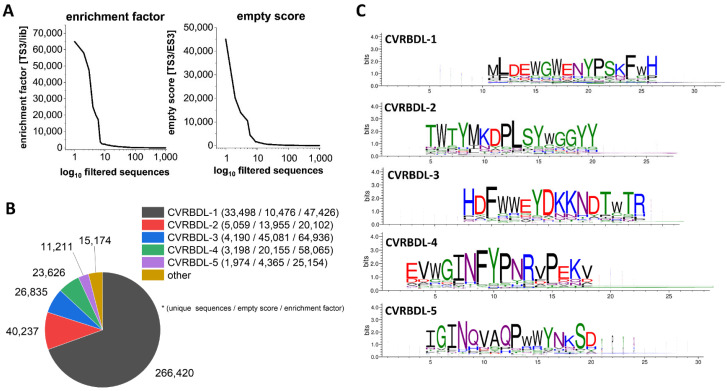
NGS sequence filtering and clustering reveals five main sequence motifs. (**A**) Evaluation of the enrichment factors (TS3/lib) and empty scores (TS3/ES3) of the first 1000 sequences as ranked by their enrichment factor or empty score, respectively. Sequences were filtered according to their enrichment on the target (lib > TS1 > TS2 > TS3), their frequency in the target selection as compared to the empty selection (TS1 > ES1; TS2 > ES2; TS3 > ES3) as well as their frequency decrease when the target selection was subjected to a surface without target (TS2 > DC2; TS3 > DC3). (**B**) Cluster distribution of the filtered sequences after three consecutive rounds of cluster extension and merging. The pie chart depicts the total number of sequences that were assigned to one cluster including multiple occurrences of identical sequences. The legend shows the number of unique sequences that were assigned to each cluster. Additionally, for each main sequence of the cluster, the specific empty score and enrichment factor is listed. (**C**) Conserved cluster motifs resulting from cluster assignments as shown in (**B**) derived by Hammock clustering software.

NGS sequencing of phage-isolated ssDNA yielded about 500,000 to 600,000 sequence reads per sample. In order to identify sequences that were enriched during phage display selection due to specific binding to the target protein, the TS3 sequences were filtered according to their enrichment on the target during the selection (lib < TS1, TS2 < TS3), their frequency in the target selection as compared to selections missing the target protein (TS1 > ES1, TS2 > ES2, TS3 > ES3), and the relative frequency decrease when a target-selection-derived sequence was subjected to a surface without target (TS2 > DC2, TS3 > DC3). In total, 90,000 unique sequences were identified that passed the filtering process. Analysis of the sequence enrichment (enrichment score [TS3/lib]) revealed that 18 unique sequences were enriched by a factor of 1000 or more. More than 50% of the total number of all sequence reads that passed the filter are represented by these 18 variants (Figure 2A). Additionally, 14 of the 18 sequences were identified with an ES of at least 1000 (empty score [TS3/ES3]), which underlines the target specificity of these sequences.

Next, the sequences were clustered according to their sequence homology in three consecutive rounds of cluster alignment and extension using Hammock clustering software (37). Clustering resulted in 16 clusters containing 57,712 unique and 383,503 total sequences, where ~96% of total sequence reads that passed the filter were assigned to five main clusters. The clusters were named CVRBDL-1 to -5, corresponding to the descending order of their total number of assigned sequence reads. The largest proportion by far, with over 70% of total sequence reads, was assigned to CVRBDL-1, followed by 10, 7, 6 and 3% for CVRBDL-2 to -5 (Figure 2B). Based on the sequence alignments, motifs were generated for each of the main clusters (Figure 2C), revealing five highly conserved sequence motifs, including amino acid residue variations, which can be used for potential sequence optimization steps. The amino acid residues with the highest occurrence at each position determined the peptide sequence of the peptides together with three C-terminally introduced arginines for enhanced solubility and C-terminal amidation: CVRBDL-1: MLDEWGWENYPSKFWHRRR-NH2; CVRBDL-2: WTYMKDPLSYWGGYYRRR-NH2; CVRBDL-3: HDFWWEYDKKNDTWTRRRR-NH2; CVRBDL-4: EVWGINFYPNRVPEKVRRR-NH2; CVRBDL-5: IGINQVAQPWWYNKSDRRR-NH2.

### 3.3. Affinity of CVRBDL Peptides towards the SARS-CoV-2 RBD

To verify whether the compounds showed affinity towards the selection target, the biotinylated SARS-CoV-2 RBD, as already used for phage display selection, was immobilized on a streptavidin-derivatized sensor surface and the compounds CVRBDL-1 to -5 were injected as analytes in a multicycle SPR experiment, which was subsequently evaluated by steady-state analysis.

**Figure 3:**
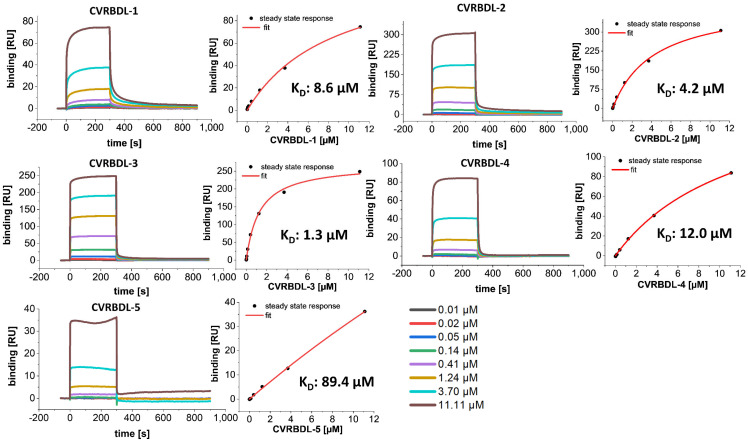
SPR affinity measurement of compounds CVDRBDL-1 to -5 on the immobilized SARS-CoV-2 RBD selection target. The C-terminally biotinylated SARS-CoV-2 RBD was immobilized on a streptavidin-derivatized sensor surface in a T200 SPR device (SA, Cytiva, USA). The compounds were injected for 300 s with 30 µL/min in a range of 0.01 to 11.11 µM in 10 mM HEPES (pH 7.4), 150 mM NaCl, 3 mM EDTA, 0.005% (*v*/*v*) Tween-20. Surface regeneration in between cycles was performed using 30 s injections of an acidic regeneration cocktail (pH 5.0), (0.15 M oxalic acid, 0.15 M H_3_PO_4_, 0.15 M formic acid, 0.15 M malonic acid). The K_D_ was determined by steady state fit as follows: CVRBDL-1: 8.6 µM ± 1.3 µM; CVRBDL-2: 4.2 µM ± 0.38 µM; CVRBDL-3: 1.3 µM ± 0.12 µM; CVRBDL-4: 12.0 µM ± 0.87 µM; CVRBDL-5: 89.4 µM ± 32.0 µM. K_D_ evaluation was performed using the Biacore T200 data evaluation software v. 3.2 with offset set to zero.

All of the tested peptides showed affinity towards the immobilized SARS-CoV-2 RBD in the low µM range as determined by steady-state analysis. As typical for peptides interacting with a folded binding partner, a transient binding behavior was detected for most of the compounds (Figure 3). However, the transient binding proportion CVRBDL-1 and -2 showed a partially slower complex decay, which can be recognized by a non-mono-exponential complex decay during the dissociation phase. This suggests that multiple binding sites for these peptides may exist, which would be a reasonable explanation for these sequences to be assigned as main sequences of the most prominent clusters as shown previously (Figure 2). Interestingly, the lowest affinity constant and therefore highest complex stability was identified for CVRDBL-3 with a K_D_ of 1.3 µM, showing a ~70-fold higher affinity than the least stable interaction with CVRBDL-5. 

**Figure 4:**
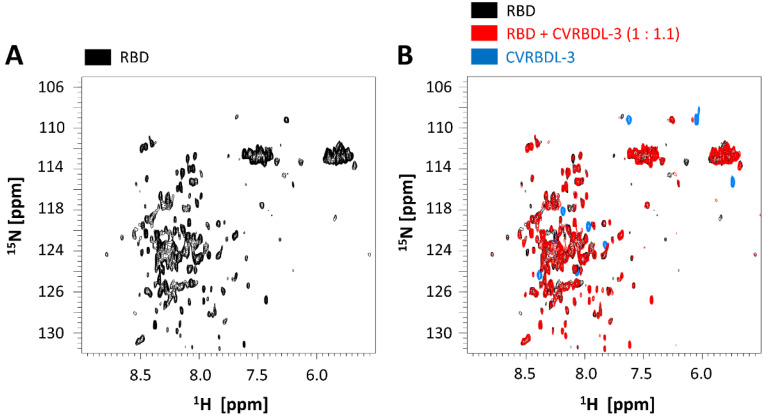
Two-dimensional ^1^H-^15^N HMQC spectra (at 1.2 GHz proton frequency) of the RBD domain (natural abundance) in absence (**A**) and presence (**B**) of the peptide CVRBDL-3. (**A**) Reference spectrum of the RBD alone. (**B**) The spectrum of the RBD domain alone (black) is overlaid with the spectra of the RBD in presence of the peptide (red), as well as the isolated peptide (blue).

Further, binding properties of the peptide CVRBDL-3 to the RBD domain were investigated by solution NMR spectroscopy. Two-dimensional ^1^H-^15^N HMQC (Heteronuclear Multiple Quantum Coherence) spectra of an 0.85 mM RBD domain sample (unlabeled, natural abundance) were recorded using a Bruker AvanceNeo 1.2 GHz spectrometer (Figure 4), as described in the Material and Methods Section. Figure 4A shows the ^1^H-^15^N correlation spectrum of the RBD domain. Roughly, every resonance in the spectrum corresponds to one amino acid residue of the protein sequence, with the nitrogen resonance correlated to the proton resonance of the backbone amide group. Therefore, the HMQC spectrum serves as a “fingerprint” of the protein. Because of the natural abundance of the ^15^N isotope of only 0.37%, the “NMR active” concentration of the 0.85 mM RBD sample corresponds to about 3 µM. Therefore, presumably only the most intense (most dynamic) residues of the RBD domain are visible in the spectra, while the more rigid regions stay below the detection threshold and thus remain invisible. 

Next, the CVRBDL-3 peptide was added to the RBD domain at a molar ratio of about 1.1:1 (peptide: RBD domain). The resulting spectrum showed decreased intensities for several resonances of the RBD domain (Figure 4B, red spectrum), compared to the reference spectrum of the unbound RBD (black). A possible explanation is that residues involved in the binding become more rigidified upon peptide interaction, leading to decreased intensities, as a result of faster transverse relaxation. A second reference spectrum of the peptide only was recorded (blue, Figure 4B). Interestingly, the resonances of the peptide (natural abundance, 1.1 higher concentration than the RBD domain) disappear in the spectra of the RBD plus peptide sample (Figure 4B, red spectrum), clearly indicating binding. Presumably, the peptide binds to the more rigid (invisible) region of the RBD and therefore becomes invisible as well (as it assumes the same NMR relaxation properties as the invisible part of the RBD).

### 3.4. Inhibition of the SARS-CoV-2 S1S2 Spike Protein Interaction with hACE2

Phage display selection was combined with NGS to identify peptides that target the SARS-CoV-2 RBD with medium to low µM affinities. To further verify whether the CVRBDL peptides are able to interfere with the association of the spike protein towards hACE2, SPR-based inhibition assays were performed. hACE-fc was immobilized on a protein A-derivatized sensor surface and the monomeric SARS-CoV-2 S1S2 protein (ectodomain of the spike protein) was injected as an analyte together with or without a 100-fold molar excess of one of the CVRBDL peptides.

**Figure 5:**
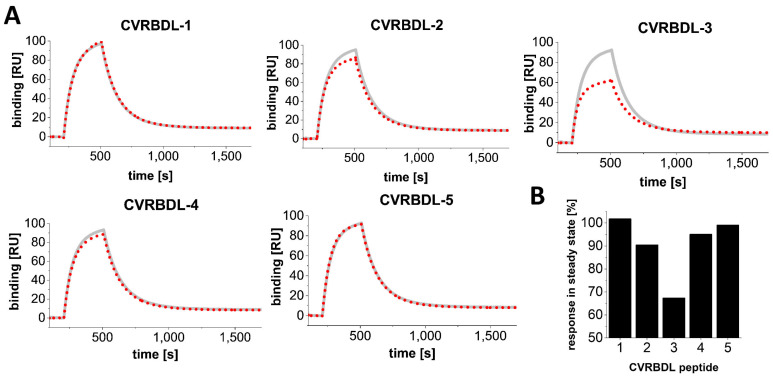
SPR inhibition assay of SARS-CoV-2 S1S2 spike interaction to hACE2 with and without compounds CVRBDL-1 to -5. (**A**) hACE2-fc was immobilized on a protein A/G derivatized sensor surface in a T200 SPR device (Cytiva, USA). 50 nM SARS-CoV-2 S1S2 was injected with (red dotted line) or without (solid grey line) 5 µM of compounds CVRBDL-1 to -5. Prior to each injection of S1S2 with compound, a separate injection without compound was performed. All injections with compound were referenced with compound-only injections on the hACE2 immobilized surface. Injections were performed with a flow rate of 30 µL/min in 10 mM HEPES (pH 7.4), 150 mM NaCl, 3 mM EDTA, 0.005% (*v*/*v*) Tween-20. (**B**) Relative steady state responses of the injections with compound as detected 270 s after injection start. CVRBDL-3 shows the strongest inhibitory effect with a steady- state reduction of ~33% as compared to SARS-CoV-2 S1S2 injection without compound.

Three out of five peptides (CVRBDL-2, -3 and -4) were able to interfere with association of the monomeric S1S2 spike protein to hACE2 in our experimental setup, which can be recognized by a reduction in the binding signal of the S1S2 protein to hACE2 in the steady-state phase upon compound addition (Figure 5A). Here, CVRBDL-3 showed the strongest inhibitory effect by a steady-state reduction of ~33% as compared to the setup without compound. CVRBDL-2 and -4 reduced the steady-state signal only by ~10 and 5%, respectively (Figure 5B). To verify whether the effect observed for CVRBDL-3 was concentration-dependent, compound concentrations in the range of 6.4 nM to 100 µM were applied to the inhibition setup. 

**Figure 6:**
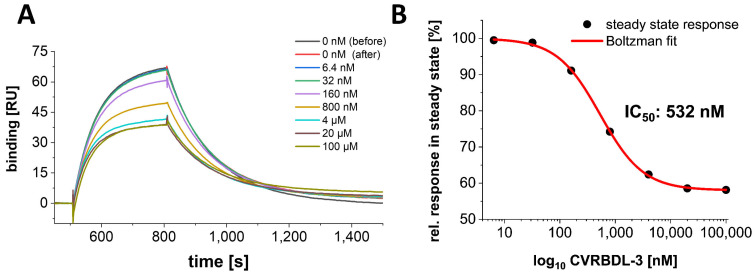
Concentration dependency of the inhibitory effect of CVRBDL-3 on the SARS-CoV-2 S1S2 interaction with hACE2. (**A**) hACE2-fc was immobilized on a protein A/G derivatized sensor surface in a T200 SPR device (Cytiva, USA). 50 nM SARS-CoV-2 S1S2 was injected with or without 6.4 nM to 100 µM CVRBDL-3. Injections without compound were performed prior and after the concentration row to demonstrate reproducibility of the S1S2 binding level. All injections with compound were referenced with the corresponding concentration of compound only injections on the hACE2 immobilized surface. Injections were performed with a flow rate of 30 µL/min in 10 mM HEPES pH 7.4, 150 mM NaCl, 3 mM EDTA, 0.005% (*v*/*v*) Tween-20. (**B**) Relative response in steady as detected at t = 750 s. The inhibitory effect of CVRBDL-3 saturates at ~58% of the S1S2 binding level without compound. The IC_50_ was determined by a Boltzman sigmoidal fit to be 532 nM (OriginPro, OriginLab, USA).

When CVRBDL-3 is applied with increasing molar ratios concerning the SARS-CoV-2 S1S2 protein, a concentration-dependent inhibition of the S1S2-hACE2 complex formation can be observed (Figure 6A). Surprisingly, the compound was not able to inhibit the complex formation completely, which can be attributed either to an insufficient sterical inhibition of the hACE2 accessibility or to an inherent heterogeneity of the S1S2-hACE2 complex. The latter has already been described by us previously [42], where we identified the S1S2-hACE2 complex formation as undergoing a two-state reaction binding behavior. In this context, the compound CVRBDL-3 is able to block the primary association of the RBD with hACE2 but possibly not the secondary state transition, which is inherent to the already formed complex. However, although CVRBDL-3 is not able to completely suppress the spike protein association to hACE2, the inhibitory effect can be characterized with an IC_50_ of 532 nM by Boltzman sigmoidal fitting, showing a maximum inhibitory saturation level of ~58% (Figure 6B). 

### 3.5. Tail-To-Tail Construct Design: CVRBDL-3_3

**Figure 7:**
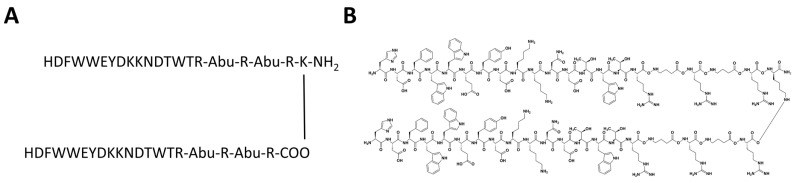
Amino acid single letter sequence and Natta projection of peptide construct CVRBDL-3_3. CVRBDL-3_3 was created by C-terminal attachment of two CVRBDL-3 sequences via the side chain primary amino group of a C-terminal introduced lysine. Each of the peptides carries a C-terminal [-Abu-R-Abu-R-] spacer region for increased binding flexibility and spacing of binding regions (Abu = γ-amino butyric acid). (**A**) Single letter amino acid CVRBDL-3_3 sequence. (**B**) Natta projection of the CVRBDL-3_3 sequence.

In order to further increase the compound affinity towards the SARS-CoV-2 spike protein, we created a tail-to-tail construct of the compound CVRBD-3 leading to a divalent compound. This construct, named CVRBDL-3_3, consists of a lysine with two CVRBDL-3 molecules covalently linked with their C-termini to the α- and ε-amino groups of the lysine via linkers each consisting of γ-aminobutyric acid (Abu) and arginine (Figure 7). The tail-to-tail connection was expected to retain the availability of the N-termini of the CVRBDL-3 moieties, which was also exposed during phage display selection as the N-terminal extension of the M13-phage coating protein pIII. 

### 3.6. CVRBDL-3_3 Shows Increased Affinity for the CoV-2 Spike Protein Compared with CVRBDL-3

**Figure 8:**
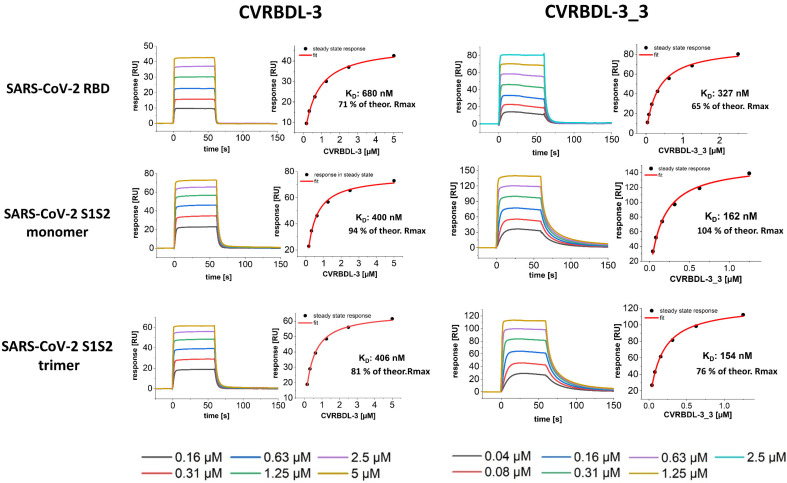
SPR affinity measurement of CVRBDL-3 and CVRBDL-3_3 on the SARS-CoV-2 RBD, S1S2 monomer and trimeric spike constructs. The spike constructs were immobilized on a polycarboxylate matrix by coupling via primary amino groups with sulfo-NHS ester at pH 5.5 (200 HCM, Xantec, GE). Measurements were performed in 10 mM HEPES (pH 7.4), 150 mM NaCl, 3 mM EDTA, 0.005% Tween-20 solution in a T200 device (Cytiva, USA). Six concentrations of each compound were injected for 60 s with a 1:1 serial dilution, with5 µM being the highest concentration for CVRBDL-3 and 2.5 µM for CVRBDL-3_3. KD evaluation was performed using Biacore T200 data evaluation software v. 3.2 with an offset of zero. The theoretical Rmax was calculated based on the molecular weight ratio of the interactants and the immobilization level after quenching.

We compared the affinity of the single peptide CVRBDL-3 and the tail-to-tail combination CVRBDL-3_3 to the poly-His tagged spike protein constructs of SARS-CoV-2 RBD, S1S2 monomer and the S1S2 trimer using SPR analysis. SARS-CoV-2 RBD, S1S2 monomer and S1S2 trimer were immobilized via primary amino group coupling on a polycarboxylate sensor surface and the compounds were injected in a multicycle experiment using concentrations in the range of 0.04 to 5 µM. The subsequent evaluation was performed by steady-state analysis (Figure 8). 

The interaction of CVRBDL-3 towards the SARS-CoV-2 RBD was identified to be ~two-fold more affine than for the original biotinylated selection target (K_D_: 680 nM vs. 1.3 µM, respectively), whereas slightly higher affinities were determined for the S1S2 monomer and the trimeric S1S2 (K_D_: 400 and 406 nM, respectively). As already observed during affinity measurement on the selection target protein, CVRBDL-3 shows again a transient binding behavior for all of the tested spike protein constructs. Additionally, the identified R_max_ was found to be lower than the theoretical R_max_ for all measurements, which indicates a 1:1 binding stoichiometry. Notably, the CVRDBL-3 binding is not influenced when the RBD is presented in the context of the monomeric and trimeric spike ectodomain. This implies that the interaction of CVRBDL-3 with the RBD binding epitope is not significantly disturbed by the RBD switch between “up-“ and “down-“ conformations. 

CVRBDL-3_3 shows increased binding affinity as compared to the single peptide alone. The complex stability increased by a factor of ~2 towards the RBD (K_D_: 327 nM) and by a factor of ~2.5 towards the monomeric and trimeric S1S2 (K_D_: 162 and 154 nM, respectively). The binding kinetics changed for CVRBDL-3_3 as compared to CVRBDL-3 from a transient binding mode with fast dissociation to a more stable complex with partially slower dissociation. However, the kinetics cannot be described with a 1:1 stoichiometric Langmuir binding behavior, indicating a distinct binding mode. Moreover, the bivalent analyte model was not able to sufficiently describe the interaction’s heterogeneity. A possible explanation could be that the aforementioned continuous position switching between ‘up’ and ‘down’ conformations of the SARS-CoV-2 RBD does not influence the interaction with CVRBDL-3 on the one side, but potentially restricts the association of CVRBDL-3_3 on the other site. This interference possibly hinders the assignment of a clear kinetic model to the observed sensograms. However, since the binding kinetics of CVRBDL-3_3 appears almost identical for monomeric and trimeric S1S2, a simultaneous binding of two RBD located epitopes with an increased avidity seems implausible, leaving room for further optimization of the linker length. 

### 3.7. CVRBDL-3 and CVRBDL -3_3 Show High Affinity for the B.1.1.7 Mutant Spike Trimer and Bind the SARS-CoV (2002) and SARS-CoV-2 “Closed” RBD Trimer with Reduced Affinity

We have previously shown that the compounds CVRBDL-3 and -3_3 show high affinity with nM binding constants towards the SARS-CoV-2 RBD, S1S2 monomer and trimer. Here, we additionally verify whether the binding specificity of the compounds is limited to the “wt” of SARS-CoV-2 spike protein or can also be extended to mutant versions, where some have been found to show enhanced affinity for the cellular receptor hACE2. Therefore, we analyzed the binding affinity of the compounds towards the SARS-CoV-2 B.1.1.7 S1S2 trimeric spike mutant (α-variant) as a model for other emerging variants of the spike protein. In total, seven amino acid sequence positions differ between SARS-CoV-2 “wt” spike protein and the B.1.1.7 variant, whereas four mutations are located at intermolecular interaction sites of the subunits and one mutation, namely N501Y, is found in the RBM of the RBD [43]. This amino acid residue substitution has been shown to increase the binding affinity for the hACE2, which may be one of the reasons for the observed four to six-fold increase in transmissibility in cooperation with other mutations that do not directly interfere with receptor binding but enhance binding e.g., by increasing RBD flexibility such as D614G [44,45,46]. 

**Figure 9:**
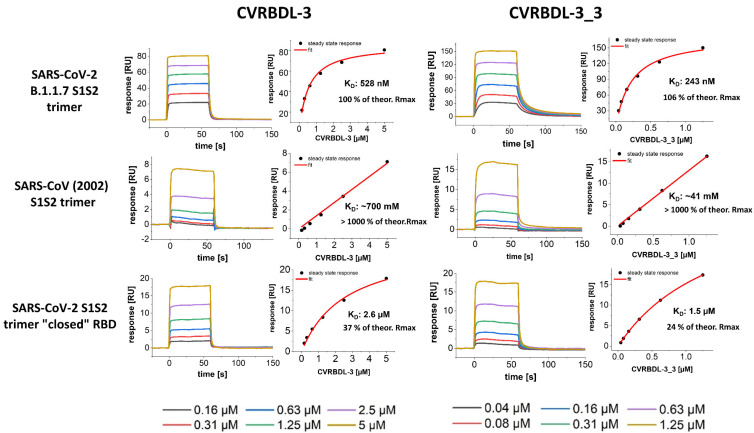
SPR affinity measurement of CVRBDL-3 and tail-to-tail construct CVRBDL-3_3 on the SARS-CoV-2 B.1.1.7 trimer spike, SARS-CoV (2002) trimer and the SARS-CoV-2 “closed” RBD trimeric spike. The spike constructs were immobilized on a polycarboxylate matrix by coupling via primary amino groups with sulfo-NHS ester at pH 5.5 (200 HCM, Xantec, GE). Measurements were performed in 10 mM HEPES (pH 7.4), 150 mM NaCl, 3 mM EDTA, 0.005% Tween-20 in a T200 device (Cytiva, USA). Six concentrations of each compound were injected for 60 s with a 1:1 serial dilution, with 5 µM being the highest concentration for CVRBDL-3 and 1.25 µM for CVRBDL-3_3. K_D_ evaluation was performed using Biacore T200 data evaluation software v. 3.2 with the offset set to zero. The theoretical Rmax was calculated based on the molecular weight ratio of the interactants and the immobilization level after quenching.

The affinity measurement on the trimeric B.1.1.7 (Figure 9) shows clearly that the binding affinity of neither CVRBDL-3 nor -3_3 is influenced by the mutation sites of the B.1.1.7 variant. Similar binding constants as already identified for the SARS-CoV-2 “wt” S1S2 spike protein were identified (528 and 243 nM for -3 and -3_3), whereas the binding kinetics shows a clear transient binding mode for CBRBDL-3 and higher complex stability with slower complex decay for CVRBDL-3_3. Since the SARS-CoV 2002 RBD shares 73% sequence identity and high structural similarity with the SARS-CoV-2 “wt” RBD [47], we speculated that the compounds are also able to bind the COVID 2002 trimeric spike protein with high affinity. 

The affinity measurements, however, show that CVRBDL-3 and -3_3 do not have any affinity for this construct (both K_D_’s in high mM range). Next, we verified the binding affinity towards the “closed” SARS-CoV-2 trimer [48]. For construct design, the spike protein was manipulated by the replacement of amino acid residues at positions 383 and 985 with cysteines in order to form a disulfide bond that fixes the RBD in its “down” position. Hence, the relevant positions for interaction with hACE2 are masked in this construct. When the compounds’ affinities were determined for this RBD “down” fixed S1S2 construct, significantly decreased affinities were observed. Additionally, the experimental R_max_ reached only 32 and 24% of the theoretical R_max_ for CVRBDL-3 and -3_3, respectively. 

### 3.8. CVRBD-3_3 Efficiently Inhibits the Spike-hACE2 Complex Formation and Displaces the Spike Protein from the Pre-Formed Complex

The tail-to-tail combination of the two CVRBDL-3 peptides achieved (CVRBDL-3_3) increased binding affinities and decreased dissociation rates for the different SARS-CoV-2 constructs. Moreover, CVRBDL-3_3 retained its target specificity and the high binding affinity towards the SARS-CoV-2 B.1.1.7 mutant spike protein. Here, we compared CVRBDL-3 and CVRBDL-3_3 concerning their ability to (i) inhibit complex formation of the SARS-CoV-2 S1S2 monomer with hACE2 and (ii) displace the spike protein from the pre-formed complex, consisting of the S1S2 monomer and hACE2. Two experimental setups allowed a complete evaluation of the compound’s effectiveness in either pre- or post-complex formation conditions. 

We injected constant concentrations of the monomeric S1S2 spike protein with or without increasing CVRBDL-3_3 concentrations on the immobilized hACE2 receptor. 

**Figure 10:**
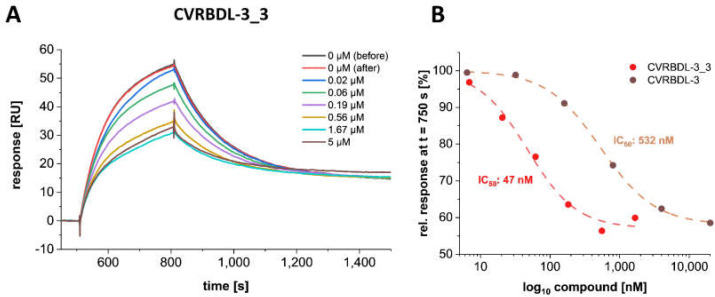
CVRBDL-3_3 much more efficiently inhibits the SARS-CoV-2 S1S2 spike protein interaction with hACE2 as compared to CVRBDL-3. (**A**) hACE2-fc was immobilized on a Protein A/G- derivatized sensor surface in a T200 SPR device (Cytiva, USA). 50 nM SARS-CoV-2 S1S2 was injected with or without 6.4 nM to 100 µM CVRBDL-3_3. Injections without compound were performed prior and after the concentration row to verify reproducibility of the S1S2 binding level. All injections with compound and spike protein were referenced with the corresponding concentration of compound only injections on the hACE2 immobilized surface. Injections were performed with 30 µL/min in 10 mM HEPES (pH 7.4), 150 mM NaCl, 3 mM EDTA, 0.005% (*v*/*v*) Tween-20. (**B**) Relative response in steady state as detected 270 s after injection start (for CVRBDL-3 see Figure 6). The inhibitory effect of CVRBDL-3 and CVRBDL-3_3 saturates at ~58% of the S1S2 binding level without compound. The IC_50_ was determined by Boltzman sigmoidal fit to be 532 nM (OriginPro, OriginLab, USA).

As already observed for CVRBDL-3, CVRBDL-3_3 also shows a concentration-dependent reduction of the SARS-CoV-2 S1S2 association towards hACE2 (Figure 10A). Interestingly, the inhibition saturates at 58% of the binding level without compound, which is identical with the saturation level that was previously identified for CVRBDL-3. Evaluation of the IC_50_ (Figure 10B) shows that the inhibition efficiency of complex formation increased by a factor of ~11 as compared to CVRBDL-3 (532 nM as compared to 47 nM for CVRBDL-3 and CVRBDL-3_3, respectively). 

Next, we analyzed the effectiveness of CVRBDL-3 and -3_3 in displacing SARS-CoV-2 S1S2 monomers from the pre-formed complex with hACE2. For this assay, a constant concentration of monomeric S1S2 was injected on pre-immobilized hACE2 for all injection cycles. During dissociation, increasing concentrations of the compounds were applied to the pre-formed complex.

**Figure 11:**
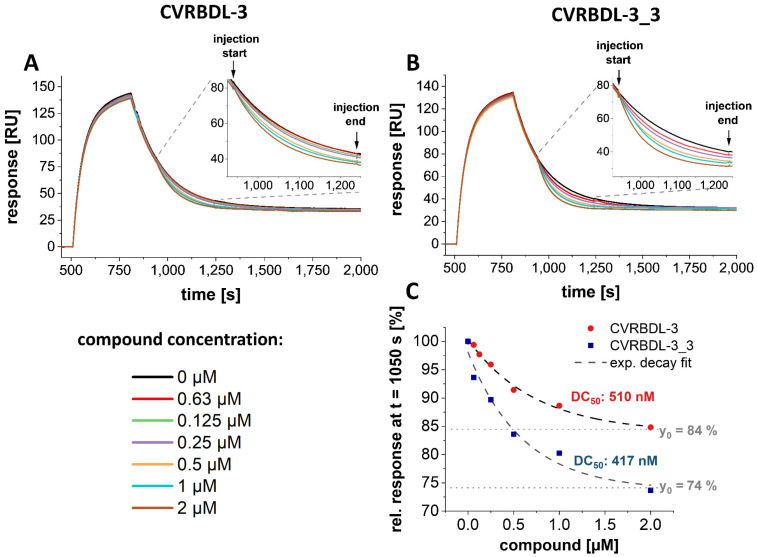
CVRBDL-3 and CVRBDL-3_3 displace hACE2 from the pre-formed complex with SARS-CoV-2 S1S2. (**A**,**B**) hACE2-fc was immobilized on a Protein A/G-derivatized sensor surface in a T200 SPR device (Cytiva, USA). For all samples, 50 nM SARS-CoV-2 S1S2 was injected for 300 s with 30 µL/min. After dissociation start 300 s of CVRBDL-3 (**A**) or CVRBDL-3_3 (**B**) was injected in a concentration range of 62.5 nM to 2 µM within six steps. All injections including compound were referenced with the corresponding concentration of compound only injections on the hACE2 immobilized surface. Injections were performed with 30 µL/min in 10 mM HEPES pH 7.4, 150 mM NaCl, 3 mM EDTA, 0.005% (*v*/*v*) Tween-20. (**C**) The response displayed in (**A**) and (**B**) at t = 1050 s of the injection without compound was set to 100%. Relative responses of the compound injections at t = 1050 s were plotted against the corresponding compound concentration. Data fitting was performed using a mono-exponential decay fit (OriginPro, OriginLab, USA). DC_50_ [nM] = compound concentration where 50% of the maximum displacement (y_0_) is reached. y_0_ [%] = offset; maximum displacement.

The sensogram course during compound injection indicates whether the compound presence accelerates the complex decay by shifting the equilibrium between hACE2 bound and unbound spike protein to the dissociated form (Figure 11A,B). For both compounds, the effect of spike protein displacement was observed by a concentration-dependent acceleration of signal reduction during the compound injection phase. When the remaining signal at t = 1050 s was plotted against the injected compound concentration, the concentration dependent effect is best described with a mono-exponential decay function (Figure 11C). Here, again CVRBDL-3_3 shows an enhanced effect as compared to CVRBDL-3. Although the concentration needed for reaching 50% of the maximum displacement just slightly differs between the two compounds (DC_50_: 510 and 417 nM for CVRBDL-3 and 3_3, respectively), the maximum displacement of CVRBDL-3_3 surpasses the one observed for CVRBDL-3 by a factor of 1.6 (y_0_: 84 and 74% for CVRBDL-3 and 3_3, respectively). 

In conclusion, both compounds show inhibitory effects when applied to pre- or post- spike-hACE2 complex formation conditions, while CVRBDL-3_3 shows enhanced inhibition as compared to CVRBDL-3.

## 4. Discussion

In the present study, we used phage display selection in combination with a unique and very efficient NGS-based data analysis strategy to identify ligands for the SARS-CoV-2 RBD. Filtering and clustering of the NGS-derived sequences using positive and negative control samples led to the identification of highly conserved cluster motifs that cover 96% of all sequences passing the analysis filter criteria and are represented by five distinct main sequences (Figure 1 and Figure 2). Further affinity analysis showed all selected peptides bind the SARS-CoV-2 RBD selection target with affinities in the low µM range, where the highest affinity was identified for sequence CVRBDL-3 with a K_D_ of 1.3 µM (Figure 3). The existence of a specific interaction between CVRBDL-3 and SARS-CoV-2 RBD was further verified by 2D-NMR using ^1^H-^15^N natural abundance correlation of the RBD (Figure 5). Here, CVRBDL-3 selectively reduced side chain resonances of the SARS-CoV-2 RBD when added at equimolar concentrations. In addition, natural resonances of the peptide were invisible when the RBD was present, suggesting a high affinity interaction between RBD and CVRBDL-3. 

Further analysis in an SPR-based experiment using the isolated SARS-CoV-2 S1S2 spike and hACE2 proteins demonstrated that CVRBDL-3 inhibits the complex formation when it is injected together with the monomeric S1S2 spike protein on the immobilized hACE2 (Figure 5). CVRBDL-3 also displaced hACE2 from the spike protein when the complex was already preformed (Figure 11). These two experimental SPR set-ups resemble physiological conditions where the virus has either not attached to the cell surface or the spike protein is already attached to the cellular receptor. A potent inhibitor targeting fusion inhibition can be expected to interfere with pre- as well as post-complex formation states. Because CVRBDL-3 is efficient in both, the targeted binding site of CVRBDL-3 on the RBD surface appears to be critical for its complex formation with hACE2 and remains at least partially accessible for CVRBDL-3 once the complex with hACE2 has been formed. 

The divalent version of CVRBDL-3, CVRBDL-3_3, had a two- to three-fold higher affinity for the SARS-CoV-2 spike protein (RBD, S1S2 monomer, S1S2 trimer) as compared to CVRBDL-3. The dissociation rates for the interaction of CVRBDL-3_3 with monomeric and trimeric S1S2 were substantially decreased for CVRBDL-3_3. Because there was no difference between the K_D_ values of CVRBDL-3_3 and monomeric and trimeric SARS-CoV-2 S1S2 spike protein (Figure 8), we conclude that the designed divalent binding mode was realized, but the increased affinity is much more likely due to the increased local concentration of the CVRBDL-3 moiety leading to decreased dissociation rates due to enhanced rebinding effects [49]. This leaves room for much more enhanced affinities by optimizing the linker length within CVRBDL-3_3 in the future. Nevertheless, CVRBDL-3_3 demonstrated increased binding affinity as compared to CVRBDL-3 in pre- as well as in post-complex formation conditions (Figure 10 and Figure 11). 

To further verify the binding specificity of CVRBDL-3 and CVRBDL-3_3, we tested their affinity towards the trimeric spike protein constructs of SARS-CoV-2 B.1.1.7, SARS-CoV (2002) and a “closed” SARS-CoV-2 spike, for which the RBD was fixed in a “down” position. CVRBDL-3 and CVRBDL-3_3 bind the B.1.1.7 mutant spike protein as efficiently as the “wt” spike trimer. This finding supports the idea that inhibitory binding sites do not necessarily overlap with the typical VOC mutational sites and thus future emerging variants may still be vulnerable to the compounds. On the other hand, the spike protein of the “old” SARS-CoV virus from the year 2002 did not show efficient binding by CVRBDL-3 and CVRBDL-3_3. This result indicates that the binding site and the observed inhibitory effect of CVRBDL-3 and CVRBDL-3_3 is specific for SARS-CoV-2 and probably not interchangeable with other SARS coronaviruses. The binding affinity, as well as the fraction of the theoretical R_max_ that is achieved towards the “closed” RBD trimeric CoV-2 constructs, is substantially decreased compared to the non-manipulated “wt” version. This result points to a decisive role of the inaccessible regions in the “closed” trimer for interaction with the compounds. 

In conclusion, affinity measurements clearly show that the CVRBDL-3 and CVRBDL-3_3 compounds target a binding site of the RBD that (i) is specific to SARS-CoV-2, (ii) is conserved in a mutant version, (iii) shows reduced accessibility in the “closed” conformation, and finally (iv) is critical for the interaction with hACE2. Phage display is an appropriate strategy for the identification of potent SARS-CoV-2 fusion inhibitors. This concept is not limited to coronaviruses only, but is transferable to other viruses, opening an option for a general and straightforward identification of drug compounds against novel emerging VOCs. The presented compound selection strategy yields small peptide compounds that are not template-protein-derived and thus are less likely prone to exhibit undesired side effects under physiological conditions. This can, for example, be expected for hACE2-template-based peptides [50,51]. The expected poor bioavailability of such L-enantiomeric peptide therapeutics may be overcome by their conversion into D-enantiomeric derivatives, for example by “retro-inverso” strategies [52] that endow all-D-enantiomeric peptide compounds with enhanced proteolytic stability [53] but conserve binding specificity and affinity. After optimization of proteolytic stability and binding affinity, future studies will first address testing of the optimized compounds in viral proliferation assays to ensure transferability of the observed effects to the cellular level.

## Data Availability

The data presented in this study are available on request from the corresponding author.

## Appendix A

**Table A1:** SARS-CoV, SARS-CoV-2 spike protein and hACE2 constructs used in the study including organism used for expression, introduced tags for multimerization and purification, molecular weight (MW) and experimental usage.

Construct Name	Expression Organism	Tag	M_W_ Monomer [kDa]	Experimental Usage
SARS-CoV-2 RBD (Acrobiosystems)	HEK293 cells	Avitag, 6xHis-tag	28.2	Phage display screening, affinity screening
SARS-CoV-2 RBD	High Five insect cells	6x His-tag	31.8	Affinity measurement with CVRBDL-3 and CVRBDL-3_3, NMR
SARS-CoV-2 S1S2-His	High Five insect cells	6x His-tag	133.13	Inhibition and displacement analysis with CVRBDL-3 and CVRBDL-3_3
SARS-CoV (2002) S1S2-His trimer	High Five insect cells	6x His-tag	132.4	Affinity measurement with CVRBDL-3 and CVRBDL-3_3
SARS CoV-2 S1S2 trimer	High Five insect cells	6x His-tag, T4-foldon	141.9	Affinity measurement with CVRBDL-3 and CVRBDL-3_3
SARS CoV-2 B.1.1.7 S1S2	High Five insect cells	6x His-tag, T4-foldon	141.6	Affinity measurement with CVRBDL-3 and CVRBDL-3_3
hACE2	HEK293-6E cells	IgG1-Fc	95.2	Inhibition and displacement analysis with CVRBDL-3 and CVRBDL-3_3
